# Extrusion of shrimp shell-polylactic acid composites: Dataset for the impact of surfactants on their morphology and thermal properties

**DOI:** 10.1016/j.dib.2021.107059

**Published:** 2021-04-29

**Authors:** John Arnold, Janelle Yen Nhi Do, Lainey C. Smith, Viktor Poltavets, Damon A. Smith

**Affiliations:** a Department of Mechanical Engineering, University of New Orleans, United States; b Department of Chemistry, University of New Orleans, United States; c Advanced Materials Research Institute (AMRI), University of New Orleans, United States

**Keywords:** Thermoplastics, Composites, Biodegradable, Green Manufacturing, Biomaterials

## Abstract

Shrimp shell waste obtained from Louisiana Gulf shrimp (Litopenaeus setiferus) was heat-treated at varying temperatures and ground into a powder by ball-milling. The powder was used with and without surface treatment with maleic anhydride or stearic acid to form shrimp shell - polylactic acid (PLA) composite granules by solution processing and mechanical grinding. These granules were used as feedstock for the extrusion of composite filaments. The dataset shows the thermal properties of the shrimp shells and the presence of covalent bonding for surface treatment with maleic anhydride. The thermal properties of the composite granules and the influence of the use of surfactants on the morphology, density, and die swell of the extruded filaments are also collected to assess their use as a manufacturing material.

## Specifications Table

**Table:** 

Subject	Chemistry, Materials Science, Manufacturing Engineering
Specific subject area	Shrimp shells, Polylactic acid, Composite, Extrusion
Type of data	Table, Image, Graph, Figure
How data were acquired	Fourier transform infrared (FTIR) spectroscopy (Nicolet IR200 FTIR), Thermogravimetric analysis (TGA) – differential scanning calorimetry (DSC) (TA Instruments SDT q600 TGA-DSC), Optical microscopy (40x-1500x Infinity Kohler Plan Inverted Microscope with 18MP Camera), Digital camera (Elmo-12F)
Data format	Raw, Analyzed
Parameters for data collection	Ball-milled shrimp shell powder was used as-is or treated with maleic anhydride. Shell powder and polylactic acid were dissolved in chloroform with or without the addition of stearic acid. The solutions were dried in a vacuum oven followed by mechanical grinding prior to thermal characterization. The granules were extruded through a round die prior to morphological characterization.
Description of data collection	Optical microscope images of the ball milled shells were acquired at 10x, 20x, and 40x magnification. FTIR spectra of the maleic anhydride and untreated shrimp shell powder were obtained using attenuated total reflectance (ATR) mode with a resolution of 4 cm^−1^. TGA-DSC data for 10 mg of composite granules with maleic anhydride treated shell powder or with the addition of stearic acid were collected at a rate of 10 °C min^−1^ in a nitrogen atmosphere.
Data source location	University of New Orleans, New Orleans, LA, USA
Data accessibility	All raw data are available in this article and the following repository: https://data.mendeley.com/datasets/7snjbpv4y5/2

## Value of the Data

• This data is useful for understanding the influence of surfactants on the morphology and thermal properties of extruded polylactic acid with shrimp shell waste included as a filler material.
• Researchers and manufacturers can use this data as guidance to determine the need for surfactant additives when developing biodegradable thermoplastics employing crustacean waste streams.
• They can be reused and combined with additional composite formulations and testing dependent on future targeted physical properties for manufactured objects.

## 1 Data Description

**Fig. 1:**
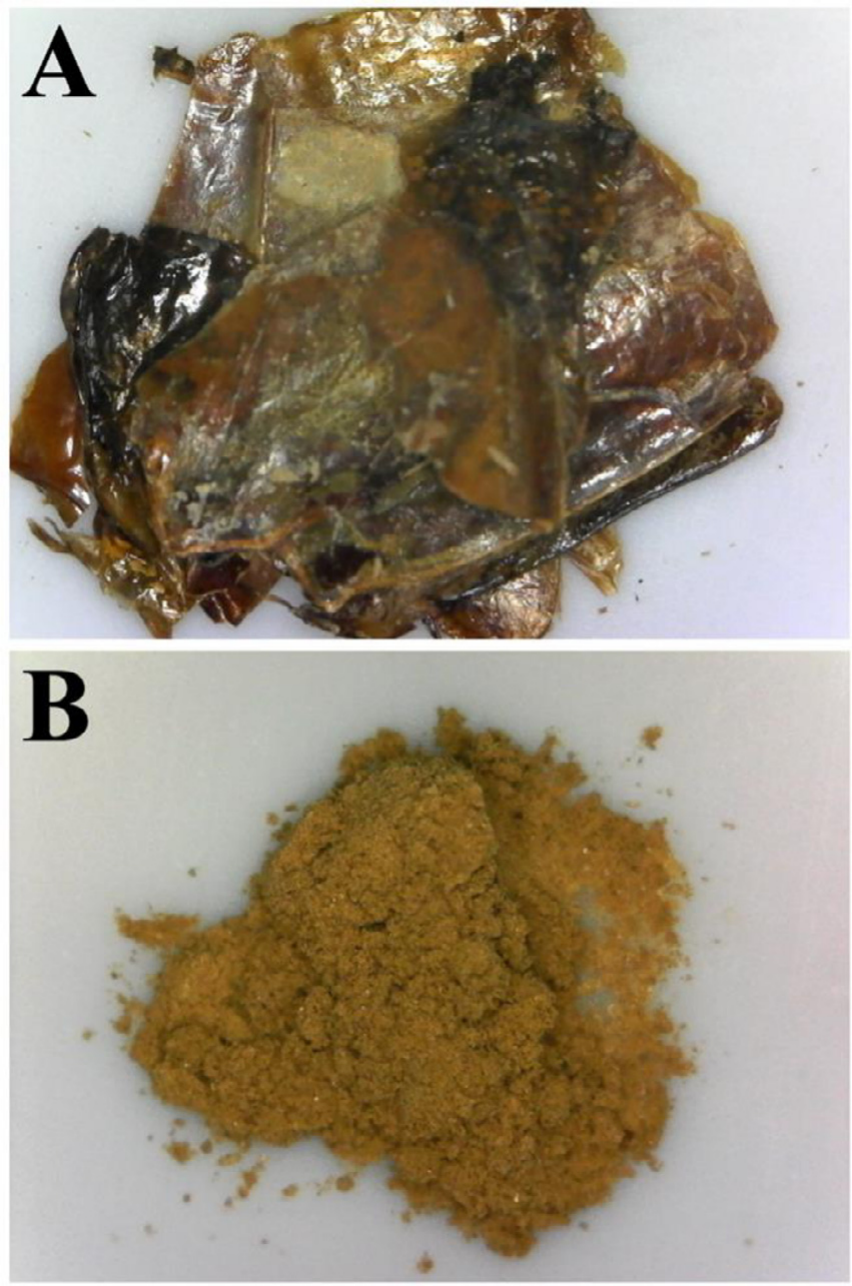
Representative photos of (A) shrimp shells dried for 2 h at 210 °C then (B) ground to a powder in a planetary ball-mill.

Fig. 1 shows representative photos of dried shrimp shells after heat-treatment (Fig. 1A) and after subsequent ball-milling (Fig. 1B).

**Fig. 2:**
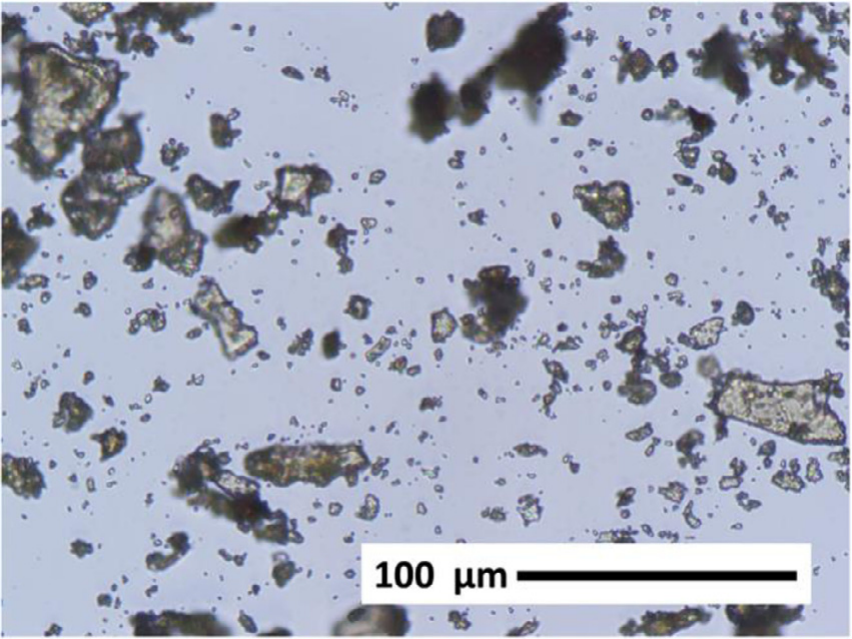
Optical microscope image at 40x magnification of shrimp shells dried for 2 h at 210 °C then ball-milled to a micron-scale powder.

Fig. 2 is an optical microscope image at 40x magnification of the shrimp shells heat-treated at 210 °C for 2 hours and ball-milled into a micron-scale powder. Additional images at 10x and 20x magnification are provided in the data repository.

**Fig. 3:**
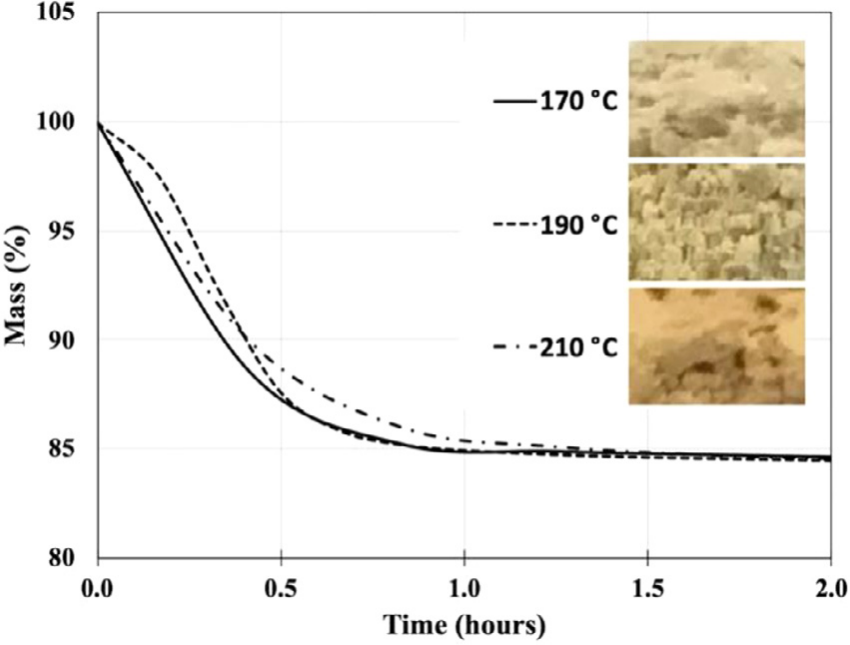
Thermogravimetric analysis (TGA) showing mass loss over a two-hour period for shrimp shells held at temperatures of 170, 190, and 210 °C. Inset: Photos of ball-milled shells for each temperature exhibiting a darkening in color with increasing temperature.

Thermogravimetric analysis (TGA) was performed to determine the heat-treatment necessary to thermally stabilize the shrimp shells for processing at extrusion temperatures. The TGA data shown in Fig. 3 indicates the mass loss for raw shrimp shells held at temperatures of 170, 190, and 210 °C over a period of 2 hours. The TGA data shows the mass loss at each temperature is approximately 15 % after 1 hour and then stabilizes after a 2-hour period. The inset of Fig. 3 shows photos displaying the darkening of the ball-milled shells with increasing heat-treatment temperature.

**Fig. 4:**
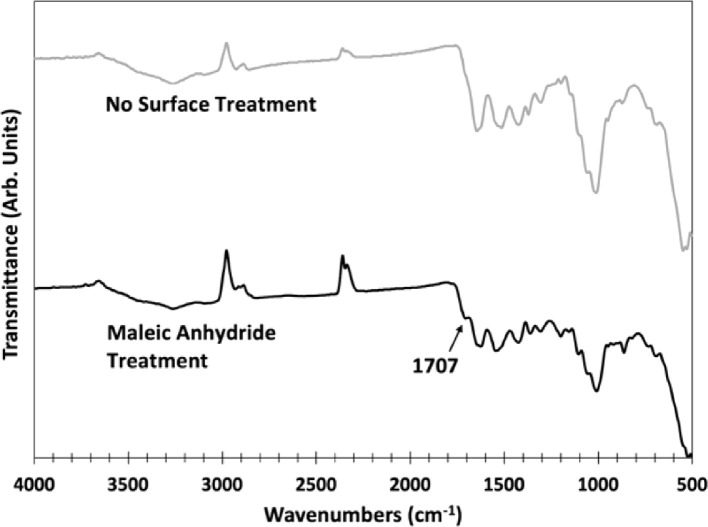
Fourier-transform infrared (FTIR) spectroscopy comparing spectra of shrimp powder with no surface treatment to powder treated with maleic anhydride (MA). The arrow points to a peak at 1707 cm^−1^ visible when treated with MA, indicative of covalent bonding between MA and chitin within the shrimp powder [1].

Fig. 4 compares the Fourier transform infrared (FTIR) spectra of ball-milled shrimp powder with no surface treatment to powder treated for covalent bonding of maleic anhydride (MA). The presence of an additional peak in the MA-treated spectra at 1707 cm^−1^ is shown in the figure, indicative of covalent bonding between MA and the carbonyl group in the chitin portion of the shrimp shells [1]. The peak is absent in the spectra of untreated shrimp shells (Fig. 4 top) as well as in the spectra of pure MA as can be seen in the NIST Chemistry WebBook database (webbook.nist.gov). The small difference in the peak position in this work 1707 cm^−1^ with 1718 cm^−1^ reported in the literature [1] can be related to the difference in protonation of the carboxylic group. Unlike the procedure used herein, the pH was adjusted to 1-2 in Ref. [1].

**Fig. 5:**
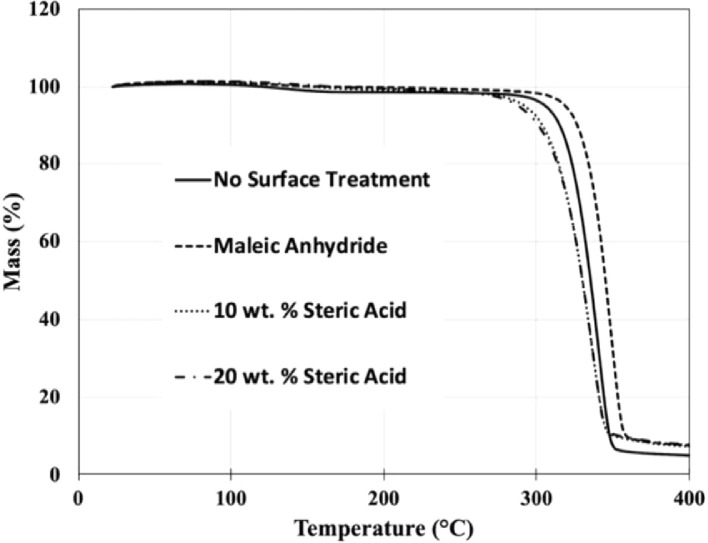
TGA data of shrimp powder-polylactic acid (PLA) composite granules with varying surface treatments showing the mass loss exhibited for temperatures up to 400 °C.

Fig. 5 shows the thermal stability of the composite granules by indicating the percent mass loss with no surface treatment, covalent bonding with MA, and with the addition of 10 or 20 wt. % stearic acid (SA) to the composite mixture. This data was collected to ensure thermally stability of the composite at the extrusion process temperatures used.

**Fig. 6:**
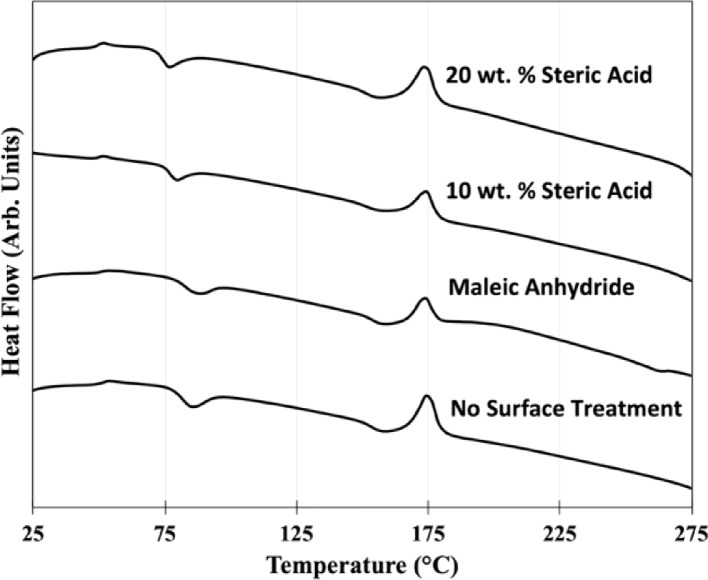
Differential scanning calorimetry (DSC) data of shrimp powder-PLA composite granules with different surface treatments.

Normalized and shifted differential scanning calorimetry (DSC) data of the composites are shown in Fig. 6. This data was collected to establish the glass transition temperature, T_G_, and the melting point, T_M_, of the composite for determination of the appropriate temperatures for manufacturing processes. Table 1 summarizes the glass transition temperature, T_G_, and the melting point, T_M_, for the various composites extracted from the DSC data in Fig. 6. DSC of the composites showed only a moderate change in the T_G_ and T_M_ for the composites for the varying surface treatments.

**Table 1:** Glass transition temperature, T_G_, and the melting point, T_M_, of composite granules with different surface treatments extracted from DSC data.

	Glass Transition	Melting
Surface Treatment	Temperature (°C)	Point (°C)
None	51.7	174.6
Maleic Anhydride	50.9	173.9
10 wt. % Steric Acid	49.4	174.2
20 wt. % Steric Acid	49.6	173.5

**Fig. 7:**
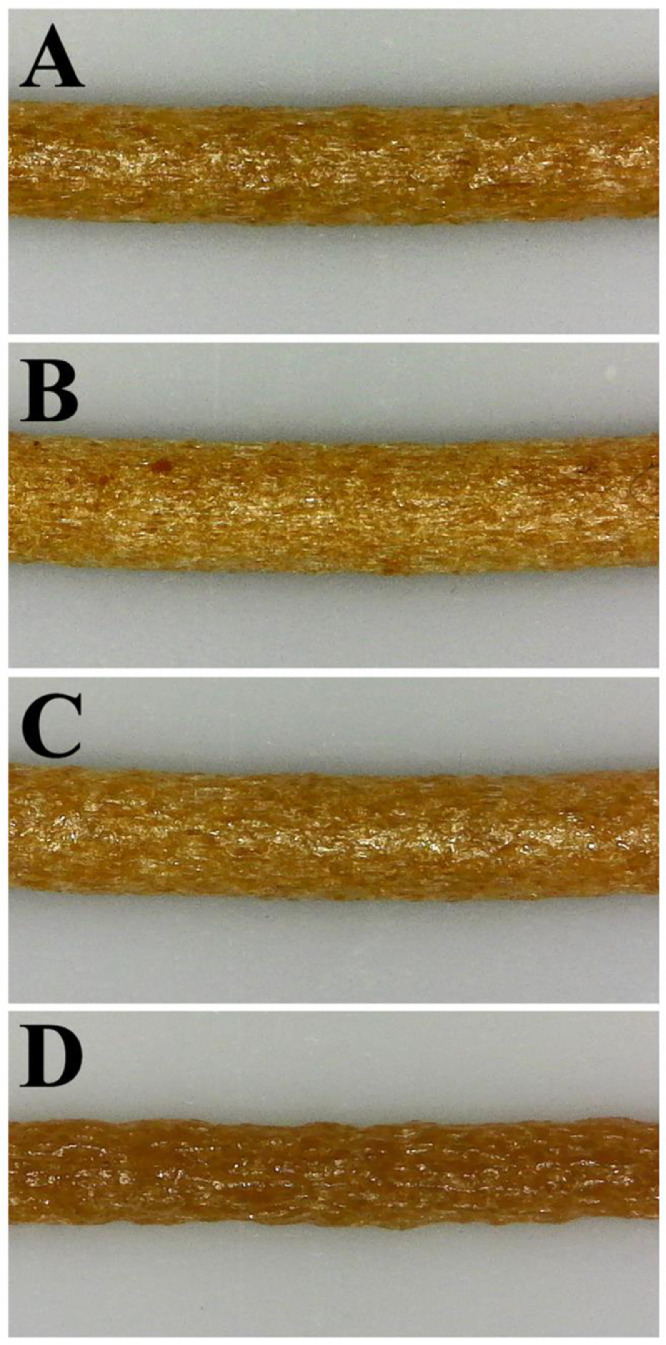
Photos of extruded shrimp powder-PLA composites with (A) no surface treatment, (B) covalently-bonded maleic anhydride, and with the addition of (C) 10 wt. % steric acid, and (D) 20 wt. % steric acid.

Fig. 7 shows photos of extruded filaments produced from the composite granules with different surface treatments. The images indicate variation in diameter and morphology with the different surface treatments. The composites initially expanded when exiting the extrusion die and then contracted to a diameter lower than the round die diameter of 1.75 mm. Table 2 shows the average of the extruded filament diameter, density, and die swell ratio for the different surface treatments. A die swell ratio of less than one was observed for all composites. Density measurements showed that the composites with MA, 10 wt. % SA, and no surface treatments were significantly lower than the density of PLA of 1.24 g cm^−1^ [2]. Composite formulations with SA added at a concentration of 20 wt. % resulted in a much higher density of 1.129 g cm^−1^. The die swell ratio is also reduced significantly to 0.49 with the additional of 20 wt. % SA.

**Table 2:** Diameter, density, and die swell ratio for shrimp powder-PLA filaments extruded through a 1.75 mm die with varying surface treatments.

Surface Treatment	Diameter (mm)	Density (g/cm^3^)	Die Swell Ratio
None	1.59 ± 0.09	0.757 ± 0.009	0.83 ± 0.07
Maleic Anhydride	1.55 ± 0.04	0.732 ± 0.003	0.79 ± 0.03
10 wt. % Steric Acid	1.55 ± 0.06	0.662 ± 0.005	0.79 ± 0.05
20 wt. % Steric Acid	1.23 ± 0.05	1.129 ± 0.007	0.49 ± 0.02

## 2 Experimental Design, Materials and Methods

### 2.1 Processing of shrimp shell-polylactic acid composites

Raw shell waste produced from Louisiana Gulf shrimp, species Litopenaeus setiferus (Laitram, Harahan, LA), were rinsed in water, air-dried, and then heat-treated in an alumina crucible placed in a tube furnace under nitrogen flow for 2 hours at a temperature of 170 °C, 190 °C, or 210 °C. The heat-treated shells were loaded into a 45 mL stainless-steel container containing three 10 mm diameter tungsten carbide balls and ground in a planetary ball-mill (Pulverisette 7, Fritsch) at 550 RPMs for six 15 min cycles. Two different surface treatments were investigated for the shrimp shells that where ball-milled and heat-treated at 210 °C for 2 hours. A surface treatment was explored to covalently bond maleic anhydride (MA) onto the surface of the shrimp powder. The MA treatment was performed by mixing MA (99.0%, Sigma Aldrich) at a 10 wt. % concentration with the milled shell powder at 120 °C for 3.5 hours. The mixture was cooled to 50 °C and then rinsed with acetone (Certified ACS, Fisher Scientific). A treatment with stearic acid (SA) was also performed by combining SA (98%, Alfa Aesar) at 10 or 20 wt. % with the shell powder in chloroform (Certified ACS, Fisher Scientific) at a temperature of 50 °C for 1 hour under vigorous stirring.

A 100 g L^−1^ PLA - chloroform solution was made by adding PLA pellets (3D850, NatureWorks) to chloroform and stirred vigorously at 50 °C until they were dissolved. The shell powder with MA, SA, or without surface treatment was added at a 10 wt. % concentration to the PLA - chloroform solution. The solution was allowed to dry under ambient conditions for 48 hours followed by removal of residual solvent in a vacuum oven at 50 °C for 24 hours. The composite was then granulated (Minigran, Dynisco) until it could pass through a 5 mm screen. The granulated composite was dried for an additional 24 hours under vacuum at 50 °C prior to extrusion.

The shrimp shell - PLA composite granules were extruded using a single screw extrusion system (EX2, Filabot) at a temperature 5 °C above the T_M_ of the composites, approximately 180 °C. The composite was extruded through a 1.75 mm circular die at a rate of 150 cm min^−1^, which then passed through an air bath (Airpath, Filabot).

### 2.2 Characterization

FTIR spectroscopy (IR200 FTIR, Nicolet) was performed on dried shrimp powder and powder treated with MA to confirm successful bonding between the shell powder and MA. FTIR was conducted with a resolution of 4 cm^−1^. TGA and DSC (SDT Q600 TGA-DSC, TA Instruments) were done in a nitrogen atmosphere. TGA was performed on the air-dried shells at 170, 190, and 210 °C over a period of 2 hours to determine mass loss. The temperature stability of the composite granules was also analyzed by TGA up to a temperature of 400 °C at a rate of 10 °C min^−1^. The T_G_ and T_M_ of the composite granules were extracted from DSC curves taken from temperatures of 25 to 250 °C at a rate 10 °C min^−1^. Five 4 cm long filament specimens were collected at 300 cm intervals during the extrusion process for diameter and weight measurements. Three diameter measurements were made on each specimen with digital calipers and the weight was measured using a laboratory scale. The density of the filament was estimated using the measurements and the die swell ratio was calculated as the average filament diameter divided by the die area.

## Ethics Statement

This work did not involve human subjects, animal experiments, or data collected from social media platforms.

## CRediT Author Statement

**John Arnold:** Conceptualization, Methodology, Formal analysis; **Janelle Yen Nhi Do:** Conceptualization, Methodology, Formal analysis; **Lainey C. Smith:** Conceptualization, Methodology, Formal analysis; **Viktor Poltavets:** Supervision, Conceptualization, Funding acquisition; **Damon A. Smith:** Supervision, Conceptualization, Writing – review & editing, Funding acquisition, Project administssration.

## Declaration of Competing Interest

The authors declare that they have no known competing financial interests or personal relationships which have or could be perceived to have influenced the work reported in this article.
